# Supplementary material for: Serum Metabolomic Profiling Reveals Differences Between Systemic Sclerosis Patients with Polyneuropathy
Source: Int J Mol Sci. 2025 Jul 24;26(15):7133. doi: 10.3390/ijms26157133 (PMC12346654; doi:10.3390/ijms26157133)
Supplement: Supplementary file 1 [file ijms-26-07133-s001.zip › ijms-3739736-supplementary.pdf]

Supplement Table S1. *Significant fold changes and p-values when comparing systemic sclerosis patients with healthy controls. Significance cutoff FC>1.5 and p-value < 0.05 were used. Metabolites with a fold change > 2 are in bolt. Metabolite name as well as abbreviations used in the volcano plot are shown.*

Metabolite	Abbreviation	FC	p-value
Aspartic acid	**Asp**	**0.14587**	**4.32E-10**
Glutamic acid	**Gln**	**0.28049**	**8.10E-11**
Valine	**Val**	**0.29542**	**1.02E-10**
Carnitine	**Car**	**0.37034**	**4.65E-20**
Citrulline	**Cit**	**0.45145**	**0.000165**
Creatinine	Cr	0.49263	2.99E-18
4-Hydroxyproline	Hyp	0.50524	2.69E-06
Isovalerylcarnitine	Kyn	0.50877	2.94E-09
Isoleucine	Iso-C5 Car	0.5191	3.00E-05
Tryptophan	Trp	0.53146	2.89E-15
Glycine	His	0.53657	4.09E-12
Threonine	Thr	0.57203	1.29E-10
Serine	Ser	0.58283	5.61E-15
Taurine	Taur	0.58897	8.41E-09
Phenylalanine	Phe	0.59519	6.67E-16
Asparagine	Asp	0.60563	5.86E-07
Methylhistidine	MeH	0.61536	0.007815
Lysine	Lys	0.62356	4.27E-11
Butyrylcarnitine	C4 Car	0.63189	3.36E-05
Tyrosine	Tyr	0.63537	3.79E-07
Glutamine	Gly	1.6535	3.35E-11

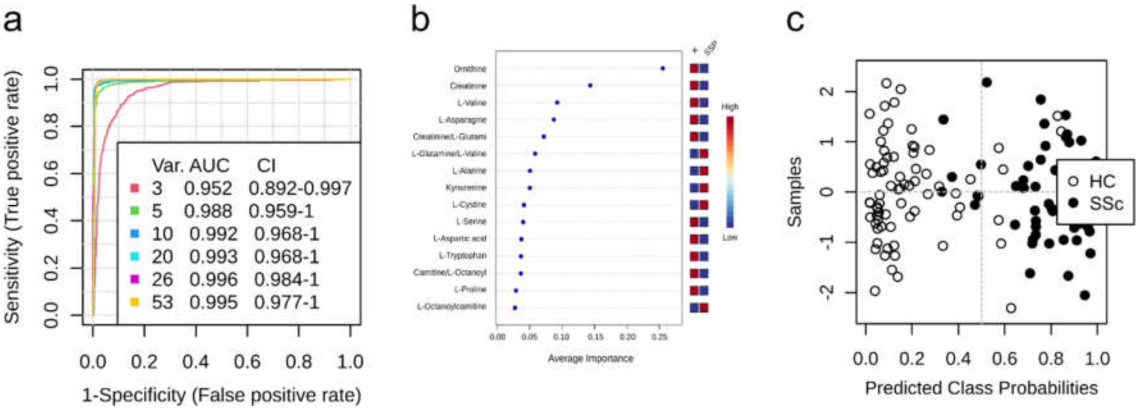

Supplement Figure S1. *Exploratory analysis of predictive models for disease classification of SSP from healthy controls (HC) using linear support vector machine. (a) ROC curves for all models with confidence intervalls (CI) were averaged or calculated from 100 cross-validations respectively. (b) Selection frequency of metabolites for the first model (using 3 variables), and (c) prediction of all patients using the first model (3 variables). Abbreviations: Var., number of variables; AUC, area under the curve.*

Supplement Table S2. *Correlation analysis of plamsa metabolites with age for healthy controls (HC) and systemic sclerosis (SSc) patients. Significant metabolites are annotated in bolt, metabolites used for prediction model added for comparison. Significance cuttoff of FDR<0.05 was used.*

Group	Metabolite	Correlation	FDR
HC	***Ornithine***	**0.39463**	**0.011227**
	Glutamic acid	**0.34365**	**0.031326**
	Leucine	**0.34032**	**0.031326**
	Creatinine	0.23296	0.17752
	Aspartic acid	-0.2266	0.1775
	Carnitine	0.1450	0.4204
	Glutamine	-0.1175	0.5328
	Valine	0.0021	0.9860
SSc	***Acetylcarnitine***	**0.4824**	**0.00122**
	Creatinine	0.17391	0.51876
	Carnitine	0.10519	0.89033
	Valine	-0.08718	0.89558
	Glutamine	-0.04801	0.94108
	Glutamic acid	0.037042	0.94108

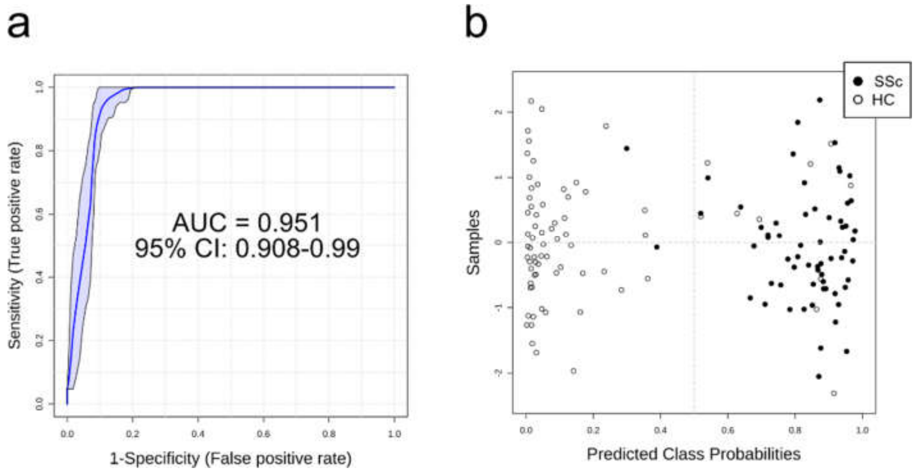

Supplement Figure S2 *Prediction model distinguishing systemic sclerosis (SSc) patients from healthy controls (HC). The model is build on linear SVM using metabolites identified by univariant analysis, however glutamic acid was left out due to its age correlation in healthy controls. (a) ROC curve and CI were calculated from 100 cross validations, average values were used for ROC plotting. (b) Class prediciton of SSP and HC, used for model building. Abbreviations: AUC, Area under the curve; CI, confdence interval.*

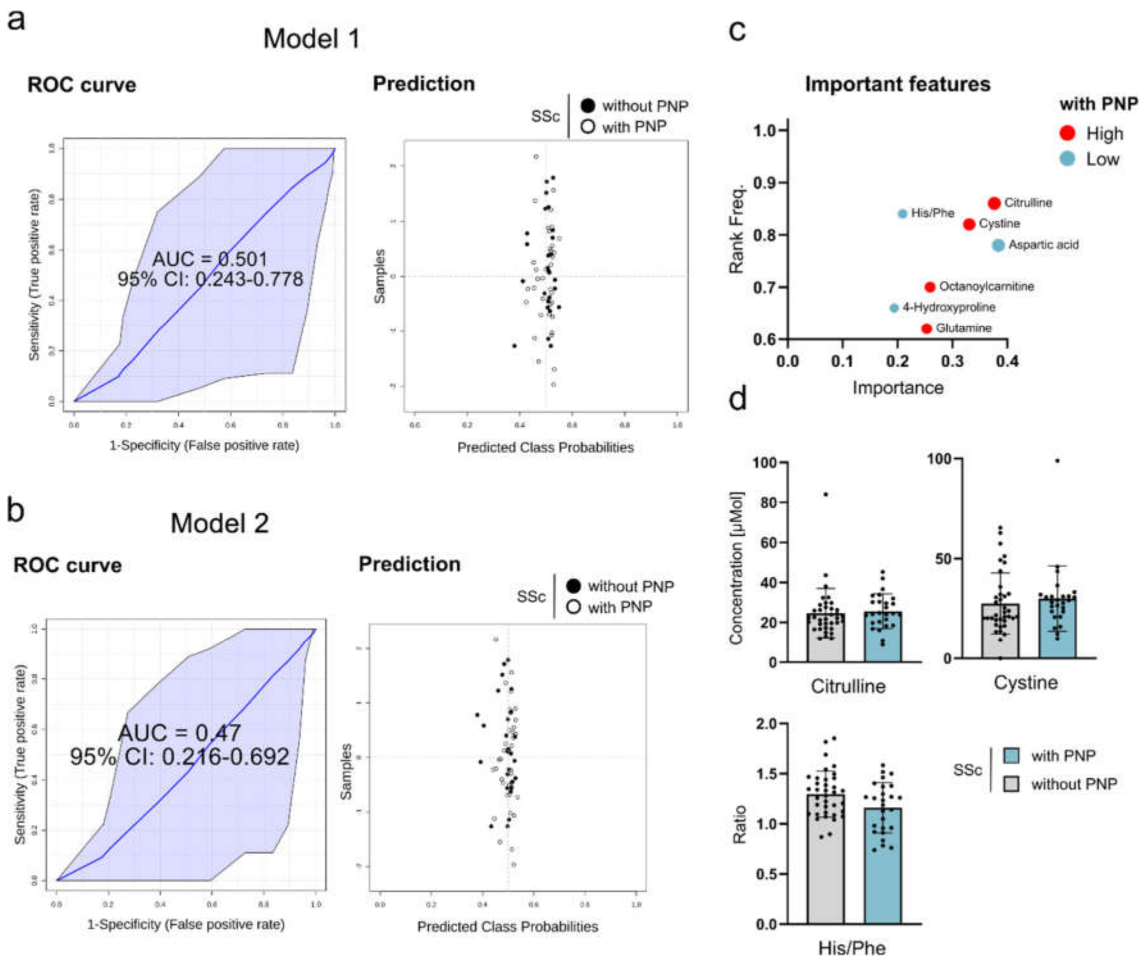

Supplement Figure S3. *Predictive models distinguishing systemic sclerosis (SSc) patients with and without polyneuropathy (PNP). Metabolites were selected based on (a) univariant significant changes, or (b) high predictive scores. Models were built on linear support vector machine, ROC curves and confidence intervals (CI) were averaged and calculated from 100 cross-validations respectively. Class prediction of samples are shown to demonstrate separation power. (c) Importance scores of the 7 highest-ranking metabolites for linear support vector machine prediction used for metabolite selection of model 2. (d) Plasma metabolite concentrations and their ratios used in model 2. Bars represent mean values of SSc patients with PNP (blue) and without (grey), individual measurements are overlaid as dots. Abbreviations: ROC, receiver operating characteristic curve; AUC, area under the curve; His, histidine; Phe, Phenylalanine.*
